# One Health Education for Children as a Catalyst for Systems Change and Climate Action in Africa

**DOI:** 10.3389/phrs.2025.1608071

**Published:** 2025-11-14

**Authors:** Wilfred Angie Abia, Richard Fomboh, Aurélie Mambap Kognoudjui, Eucharia A. Abia

**Affiliations:** 1 Agri-Food Safety and One Health Agency (AFS1HA), Yaounde, Cameroon; 2 International One Health for One Planet Education (1 HOPE) Primary and Secondary School Initiative Africa (1 HOPE PS Africa), Integrated Health for All Foundation (IHAFCam), Yaounde, Cameroon; 3 Laboratory of Pharmacology and Toxicology, Department of Biochemistry, Faculty of Science, University of Yaounde 1, Yaounde, Cameroon; 4 Cameroon Christian University Institute, Bali, Cameroon

**Keywords:** ecocentrism, education for children, climate change, networking, one health

## Abstract

**Objectives:**

This paper explores the importance of education for children and communities in promoting One Health (OH) principles and fostering a holistic understanding of the interdependencies between humans, animals, ecosystems, and climate change (CC).

**Methods:**

Literature on education’s role in promoting One Health and climate awareness amongst children was screened.

**Results:**

It highlights the role of networking and system reform in creating an enabling environment for OH education, ensuring collaboration among diverse stakeholders including educators, healthcare professionals, environmentalists, and policymakers. Furthermore, it examines the impact of CC and need for OH approach with emphasis to educate children about the consequences of environmental degradation and climate-related health risks. Through interactive and interdisciplinary educational approaches, children can become accountable to environment and become advocates for sustainable CC mitigation practices. Integrating OH principles into educational curricula equips children to address complex global challenges effectively.

**Conclusion:**

This paper advocates for a comprehensive OH education approach encompassing formal, informal learning, community engagement, and policy support. Empowering children and the public with OH knowledge supports a healthier, more sustainable planetary future.

## Introduction

The One Health concept emphasizes the interconnectedness of human, animal, and environmental health. It recognizes that the health of one species is intricately linked to the health of others and the ecosystems they inhabit [1]. This holistic approach is crucial in addressing complex global challenges, as issues such as zoonotic diseases, environmental degradation, and climate change do not exist in isolation. By understanding these interdependencies, healthier communities and a more sustainable planet can be promoted. The One Health framework encourages collaboration across various disciplines, including medicine, veterinary science, and environmental studies, fostering innovative solutions to health crises [2].

Education plays a fundamental role in instilling One Health principles in future generations. Children, as the stewards of tomorrow, must be equipped with the knowledge and skills necessary to navigate the complexities of our interconnected world [3]. Effective education can empower children to become proactive agents of change, addressing the challenges posed by climate change, biodiversity loss, and public health issues. By instilling a sense of responsibility and awareness in young learners, we can inspire a new generation of advocates for sustainable practices and holistic health [4, 5].

Integrating One Health principles into educational curricula enhances children’s understanding of the interdependencies between humans, animals, and the environment while fostering critical thinking and problem-solving skills [6]. Educational initiatives that encompass both formal and informal settings can create a comprehensive learning environment. Through hands-on experiences and interdisciplinary approaches, children can explore the implications of their actions on the planet and develop an appreciation for conservation and sustainable living [3]. This educational transformation is necessary for nurturing informed and responsible future leaders.

Networking and collaboration among diverse stakeholders are essential for effective One Health education. Educators, healthcare professionals, environmentalists, and policymakers must work together to create an enabling environment that fosters knowledge sharing and resource allocation [1, 5]. Such collaboration can lead to the development of innovative educational programs that resonate with children and their communities. By building strong networks, stakeholders can amplify their impact, ensuring that One Health principles are effectively communicated and integrated into various aspects of society [2].

Climate change poses significant threats to One Health, affecting the delicate balance between human, animal, and environmental health. The increasing incidence of climate-related health risks, such as vector-borne diseases and food insecurity, underscores the urgent need for targeted education on this topic [7]. Children must be informed about the potential consequences of environmental degradation and be empowered to take meaningful action. By understanding the interconnectedness of climate change and health, they can cultivate a sense of agency and responsibility toward their environment [4, 8].

Generally, integrating One Health principles into education is vital for fostering a healthier and more sustainable future. By promoting awareness and understanding among children, we can cultivate a generation of informed citizens equipped to tackle complex global challenges [6]. This initiative requires a concerted effort from all stakeholders, emphasizing the importance of networking, system reform, and climate change awareness in creating a holistic framework for One Health education. Through these collaborative efforts, we can inspire meaningful change and promote the wellbeing of our planet and its inhabitants.

## Methods

A narrative literature review approach was adopted to explore the role of education in promoting One Health principles among children and communities. All relevant literature was identified through online searches using databases such as Google Scholar, PubMed, and ScienceDirect, as well as grey literature from reputable institutional and organizational websites such as the One Health Commission, the World Association of Disaster and Emergency Medicine (WADEM;^1^), etc. Search terms included, but not limited to, combinations of keywords such as “One Health,” “education,” “climate change,” “children,” “ecosystems,” “zoonotic diseases,” and “planetary health.”

No strict inclusion or exclusion criteria were applied, as the objective was to gather a broad understanding of interdisciplinary perspectives and existing frameworks on One Health education for young minds. Emphasis was placed on recent publications, conceptual articles, policy documents, and reports that contribute to understanding the intersection of One Health, climate change, and education, particularly for children’s education.

The selected sources were analyzed to identify recurring themes, gaps, and strategies related to One Health education, system reform, stakeholder collaboration, and climate-related health risks. Insights from the reviewed literature were synthesized to provide vital information for integrating One Health principles into educational settings. This approach was chosen to support a comprehensive understanding of the topic and to generate practical recommendations based on existing knowledge and practices.

## Results and Discussion

### Advances in One Health Education Across Different Continents

The concept of One Health, which emphasizes the interconnectedness of human, animal, and environmental health and more, has gained significant traction globally, particularly in educational frameworks. In the United States, One Health education has been notably advanced, with numerous universities incorporating interdisciplinary curricula that address the complexities of zoonotic diseases, environmental health, and public health. Institutions such as the University of California, Davis, and the Johns Hopkins University have established dedicated programs and research initiatives that foster collaboration among veterinary, medical, and environmental science students [9]. This comprehensive approach has led to enhanced awareness and preparedness for emerging infectious diseases, exemplified by the response to COVID-19. Furthermore, an international assessment of One Health education, training, and capacity-building programs has already been documented [10].

In Europe, One Health education is advancing significantly, particularly through initiatives led by Una Europa. The European Union has recognized the importance of this interdisciplinary approach in combating health threats, fostering collaboration among veterinary, medical, and environmental professionals. Programs like the One Health European Joint Programme exemplify these efforts, integrating diverse knowledge and practices across disciplines [11].

Una Europa is at the forefront of this movement, promoting innovative academic frameworks that encourage collaborative projects to tackle health challenges. Countries such as the Netherlands and Germany are leading in the implementation of One Health curricula, inspiring students to engage actively in interdisciplinary initiatives that address complex health issues across Europe.

Additionally, the Erasmus Mundus Scholar program plays a crucial role in enhancing educational mobility. This program offers joint master’s degrees that facilitate international collaboration and cultural exchange among students. By participating in Erasmus Mundus, students gain valuable insights and experiences that further enrich their understanding of One Health principles and practices.

The One Health approach is gaining traction globally, with significant contributions from networks such as the Southeast Asia One Health University Network (SEAOHUN;^2^), the International Student One Health Alliance (ISOHA;^3^), and the One Health Learning and Advocacy Initiative (OHLAIC;^4^). SEAOHUN connects over 115 universities across Southeast Asia, fostering interdisciplinary collaboration to build a workforce equipped to tackle infectious diseases. ISOHA unites students globally, promoting awareness and education about the interconnectedness of human, animal, and environmental health. Meanwhile, OHLAIC focuses on advocacy and education, empowering communities to adopt One Health principles. Together, these organizations play a crucial role in enhancing One Health education, encouraging innovative solutions, and preparing the next-generation of professionals to address complex health challenges. Their collaborative efforts highlight the importance of interdisciplinary thinking and global partnerships in advancing health outcomes across regions.

In contrast, progress in Africa is often hindered by resource constraints and a lack of formalized educational programs in One Health. While there are existing initiatives such as the GET One Health School Club (GHSC), an initiative by the Global Emerging Pathogens Treatment Consortium (GET), and initiative to introduce the One Health approach to senior secondary students in various schools;^5^ the One Health for One Planet Education (1HOPE) Initiative for Primary and Secondary Schools in Africa (1HOPE PS Africa) that is focused on taking the One Health approache and wellbeing to every angle of the Africa; and the African One Health University Network, which aims to promote collaboration among various sectors, the implementation of One Health education in academic institutions is still nascent [12]. Many universities in Africa lack the interdisciplinary curricula and resources necessary to effectively teach One Health concepts, which is critical in addressing the continent’s unique health challenges, including endemic zoonotic diseases and the impacts of climate change on health. Consequently, there is an urgent need for increased investment in One Health education across Africa to enhance health outcomes and foster collaboration among health sectors.

### The Importance of Education in One Health

Education plays a pivotal role in promoting the principles of One Health by fostering an understanding of the interconnections between human, animal, and environmental health [13]. By integrating One Health concepts into educational curricula, educators can cultivate critical thinking and problem-solving skills in students, enabling them to comprehend the complexities of global health challenges [14]. This foundational knowledge is essential for preparing future generations to address pressing issues such as emerging zoonotic diseases and environmental degradation, which require collaborative approaches across multiple disciplines.

One Health education encourages interdisciplinary collaboration among students, educators, and professionals from diverse fields such as medicine, veterinary science, and environmental studies. Such collaboration is crucial for developing innovative solutions to multifaceted health problems [15]. By fostering a culture of cooperation and knowledge sharing, educational initiatives can help break down silos that often hinder effective responses to health crises. This interconnected learning environment not only enriches students’ educational experiences but also equips them with the tools necessary to become proactive advocates for health and sustainability [16].

Engaging children and young adults in One Health education can instill a sense of responsibility towards their communities and the planet [8]. Programs designed to educate youth about the implications of climate change, public health, and biodiversity loss can empower them to take meaningful action [17]. As these future leaders become more aware of the impact of their choices on health outcomes and environmental sustainability, they are more likely to advocate for policies and practices that promote One Health principles in their communities [18, 19]. This grassroots approach is essential for fostering a culture of health and sustainability that transcends generations.

### Conceptual Framework of One Health Education for Children

The Conceptual Framework of One Health Education for children is designed to cultivate an understanding of the interconnectedness of human, animal, and environmental health from an early age. This framework emphasizes core concepts such as ecosystems, the impact of human activities on health, and the significance of biodiversity. By utilizing age-appropriate materials and interactive learning methods—such as storytelling, hands-on activities, and multimedia resources—educators can engage children effectively, making these complex ideas relatable and fostering a sense of curiosity about their surroundings [20–22]. This framework serves as a guide for implementing One Health education for children, promoting an understanding of the interconnectedness of human, animal, and environmental health (Table 1).

**TABLE 1:** Some Conceptual frameworks of One Health Education for Children (Worldwide, 2013–2025).

Community engagement	Expected outcomes	One health actions
Involving families and communities in One Health initiatives	Increased awareness and support for One Health	- Community health fairs - Workshops for parents
Integrating knowledge from health, environment, and animal sciences	Holistic understanding of health and environment	- Collaborative projects - Workshops with experts
Designing age-appropriate content that covers One Health principles	Engaged learning and retention of key concepts	- Curriculum guides - Interactive learning modules
Providing practical activities to reinforce learning	Real-world application of concepts	- Field trips to farms or nature reserves - Science experiments
Teaching children about personal health, hygiene, and environmental stewardship	Development of healthy habits and responsible behaviors	- Hygiene campaigns - Environmental clean-up projects
Engaging local health professionals, veterinarians, and environmentalists	Enhanced understanding through expert insights	- Guest lectures - Mentorship programs
Measuring the effectiveness of One Health education programs	Continuous improvement of educational strategies	- Surveys and feedback sessions - Pre- and post-tests

### Integrating One Health Into Educational Curricula

The One Health education is gradually gaining grounds globally. The One Health Commission has relented no efforts to capture, assemble and disserminate information on One Health initiatives around the globe as a monthly “*One Health Happenings*”.^6^ The “One Health Happenings” information regularly shared would have contributed either to curriculum design or at least serve as a road map not only to know “who is who in One Health”^7^ but also to facilitate collaborations for research and/or integration of the One Health into educational curriculum. Details on various One Health education, training and capacity building initiatives have been extensively reviewed [10]. A set of strategies for the integration of one health into educational curricula has been proposed (Table 2).

**TABLE 2:** Some strategies for integrating One Health into educational curricula (Worldwide, 2022–2024).

Strategy	Description	References
Interdisciplinary Curriculum Development	Integrating One Health principles into curricula by combining subjects like biology, environmental science, health education, and social studies. This approach helps illustrate how various factors influence health outcomes	[23]
Experiential Learning Opportunities	Incorporating hands-on experiences such as field trips to farms, wildlife reserves, or health clinics. These activities allow students to observe the real-world implications of One Health and foster responsibility and agency in advocating for sustainability Experiential learning opportunities include clinics like the Knights Landing One Health Center, which provides community-centered health services integrating human, environmental, and animal health. Other similar clinics may focus on underserved areas, offering hands-on training for medical and veterinary students	[18]
Collaboration with Local Stakeholders	Engaging healthcare professionals, veterinarians, and environmental organizations in education. Guest speakers and joint projects enhance learning and build networks for future One Health initiatives Effective One Health initiatives require collaboration across various fields. A notable project involves the World Veterinary Association, World Association for Disaster and Emergency Management, World Medical Association, and the International Council of Nurses, among others	[24] https://knightslandingonehealth.com
Utilizing Technology and Multimedia	Leveraging online platforms, interactive simulations, and educational videos to make complex topics more accessible and engaging. Virtual field trips and collaborative projects expand students’ horizons and encourage exploration and discovery	[23]
Promoting Critical Thinking and PBL	Using problem-based learning (PBL) to present real-world challenges, requiring students to analyze data and propose solutions. Continuous assessment and feedback help adapt the curriculum to students’ needs, ensuring relevance and impact	[13]

### Interdisciplinary Approaches to One Health Education

One Health education emphasizes the interconnectedness of human, animal, and environmental health, advocating for interdisciplinary collaboration among various fields such as medicine, veterinary science, environmental science, and public health. This holistic approach is crucial for addressing complex health challenges, such as zoonotic diseases, which require a comprehensive understanding of the interactions between these domains [25]. Recent studies highlight the importance of integrating diverse educational backgrounds to foster effective communication and collaboration among future professionals, ultimately leading to improved health outcomes [22, 26].

The incorporation of experiential learning in One Health education programs is another key strategy that enhances the interdisciplinary approach [27]. By engaging students in real-world scenarios and collaborative projects, educational institutions can cultivate critical thinking, problem-solving, and teamwork skills [28]. Programs that facilitate joint training sessions between medical and veterinary students, for example, allow future practitioners to appreciate each other’s roles in healthcare and disease prevention [29]. Such initiatives not only enrich the learning experience but also prepare graduates to tackle the multifaceted health challenges they will encounter in their careers.

Moreover, the integration of technology and digital tools into One Health education is proving to be advantageous in promoting interdisciplinary learning [30]. Online platforms and simulation tools enable students from different disciplines to collaborate on projects, share data, and analyze health-related issues in real time [31]. This digital approach can enhance accessibility and engagement, particularly for remote learners or those in underserved areas [32]. Leveraging technology, educators can create a more inclusive and dynamic learning environment that reflects the interconnected nature of health in a globalized world.

### Networking and Stakeholder Collaboration

Networking and stakeholder collaboration are essential for enhancing One Health education, as they integrate diverse perspectives and expertise. Engaging healthcare professionals, veterinarians, and environmental organizations allows students to gain insights into real-world challenges and solutions. For instance, collaborative efforts such as guest lectures and workshops can promote interdisciplinary learning and problem-solving [33]. These interactions not only enrich the educational experience but also help students understand the complexities of health issues at the human-animal-environment interface, fostering a more comprehensive approach to health education [19, 34].

Developing strategic partnerships with community organizations facilitates experiential learning opportunities that align with One Health principles. Service-learning projects, including community health assessments and environmental conservation initiatives, enable students to apply their knowledge in practical settings [14]. Such collaborations foster a sense of community responsibility and empower students to advocate for sustainable practices within their local contexts [5]. By integrating local knowledge and resources, educators can enhance the curriculum and deepen students’ connections to their communities, making learning more relevant and impactful. An example is the AlaskaX’s “*One Health: A Ten Thousand Year-Old View into the Future*” offered by the University of Alaska Fairbanks in partnership with edX.^8^ Cediel-Becerra et al. [35] emphasized the importance of inclusion and engagement of communities in One Health practices and need for partnerships including non-academics from woman-sensitive One Health Perspective in Latin America.

Establishing robust networks of stakeholders encourages ongoing collaboration and innovation in One Health education. Educational institutions can form formal alliances with governmental agencies, non-profits, and private sector organizations to create a supportive framework for curriculum development and implementation [17]. By nurturing these relationships, educators can ensure that their programs remain relevant and responsive to current health challenges [16]. This collaborative approach not only prepares students for future careers in One Health but also contributes to a more integrated understanding of health across various sectors, ultimately benefiting society as a whole.

### Models for Creating Supportive Networks in One Health Education

The Community of Practice (CoP) model fosters a collaborative environment where individuals with shared interests in One Health education engage in continuous learning and knowledge sharing through regular interactions and discussions. The Partnership Model emphasizes formal collaborations between educational institutions, governmental bodies, and community organizations to create integrated programs and resources that address One Health issues collectively. The Networked Learning Environment (NLE) leverages digital platforms to facilitate access to diverse educational resources, promote online discussions, and connect learners and professionals across geographic boundaries, enhancing the overall learning experience and fostering innovative solutions to complex health challenges. Table 3 presents some potential models that can be exploited to create supportive networks in One Health education.

**TABLE 3:** Some models for creating supportive networks in One Health education (Worldwide, 2024).

Model	Description	References
Community of Practice (CoP)	This model fosters ongoing collaboration among individuals with shared interests in One Health. It encourages regular meetings, workshops, and discussions, allowing participants to share knowledge, experiences, and resources. CoPs enhance curriculum development reflecting real-world health challenges. An example of CoP is the Women for One Health (WfOH) Network (https://wfoh.org)	[36]
Partnership Model	This model emphasizes formal alliances between educational institutions and various stakeholders. Schools and universities collaborate with local health departments, non-profits, and private organizations to create joint initiatives addressing One Health issues, facilitating hands-on experiences. An example of a Partnership model is the One Health Initiative in the Foukouka Perfecture (http://onehealth-fukuoka-en.com) where government is sponsoring a lot of One Health focused community work in promotion/education of the public	[37]
Networked Learning Environment (NLE)	This model leverages technology to create a dynamic educational framework. It uses online platforms and collaborative tools to facilitate communication and resource sharing among educators, students, and stakeholders, connecting participants across geographical boundaries for collaboration on One Health projects. An example is the One Health Commission (https://www.onehealthcommission.org/) which is the quintessential place to find information about OH information	[16]

The examples might not have been cited in the cited articles, but adding then here is for more enphasize and credibility of this report.

### Climate Change Issues Across Various Continents of the World

One Health approach links human, animal, and environmental health, addressing climate change impacts like antimicrobial resistance spread [26]. Climate change is a global challenge that manifests differently across continents, affecting ecosystems, economies, and communities in unique ways.

In North America, climate change has led to increased temperatures, altered precipitation patterns, and more frequent extreme weather events. The United States has experienced severe droughts in the West, hurricanes in the Gulf Coast, and wildfires in California and other states, all of which are exacerbated by climate change [38]. Additionally, the melting Arctic ice is impacting indigenous communities and wildlife in Canada, highlighting the urgent need for adaptive strategies [39].

Europe is facing significant climate impacts, including rising sea levels, increased flooding, and heatwaves. The European Union has recognized the need for robust climate policies, leading to the European Green Deal, which aims to make Europe the first climate-neutral continent by 2050 [40]. However, disparities exist between northern and southern Europe, with southern countries like Greece and Italy experiencing more severe droughts and wildfires, while northern regions are seeing increased precipitation and flooding [41].

Asia is particularly vulnerable to climate change due to its vast population and diverse ecosystems. Countries like Bangladesh and Pakistan are experiencing increased flooding and cyclones, which threaten livelihoods and food security [2]. In contrast, the Himalayan region is facing rapid glacier melt, which affects water supply for millions. The Asian Development Bank has emphasized the need for comprehensive disaster risk management strategies to address these challenges [42].

Africa is disproportionately affected by climate change, with impacts such as desertification, altered rainfall patterns, and increased frequency of extreme weather events. Sub-Saharan Africa faces significant challenges in food security as agricultural productivity declines due to changing climate conditions [43]. Furthermore, the continent’s limited resources and adaptive capacity exacerbate vulnerabilities, making it imperative to invest in sustainable practices and climate resilience [44].

In South America, climate change is driving deforestation in the Amazon rainforest, which not only contributes to biodiversity loss but also exacerbates global warming [45]. Countries like Brazil are grappling with the dual challenges of promoting economic development while protecting vital ecosystems. Additionally, increasing temperatures and changing precipitation patterns threaten agriculture, particularly in the Andean regions, where smallholder farmers are highly vulnerable [46].

Australia is experiencing severe impacts from climate change, including prolonged droughts, heatwaves, and bushfires. The 2019–2020 bushfire season highlighted the devastating effects of climate change on biodiversity and human health [47]. The Australian government has been criticized for its response to climate change, with calls for more aggressive policies to reduce greenhouse gas emissions and enhance resilience [48].

### The Impact of Climate Change on One Health

Climate change significantly impacts One Health by altering the ecosystems that host pathogens and vectors, thereby influencing the transmission of zoonotic diseases. As temperatures rise and weather patterns change, species distributions shift, leading to the emergence of diseases in new regions [49]. For instance, warmer climates can expand the habitats of ticks and mosquitoes, increasing the incidence of vector-borne diseases such as Lyme disease and West Nile virus. Understanding these dynamics is crucial for developing effective surveillance and response strategies to mitigate health risks associated with climate change [50]. The OneHealthLessons.org^9^ has created a One Health educational module focused on ticks and mosquitoes, helping students understand how to prevent vector-borne diseases and grasp the interconnected nature of human, animal, and environmental health.

Moreover, climate change exacerbates environmental degradation, which in turn affects human, animal, and ecosystem health. Deforestation, land use changes, and habitat destruction can lead to increased human-wildlife interactions, heightening the risk of zoonotic disease spillover. For example, the encroachment of agricultural development into wildlife habitats has been linked to outbreaks of diseases such as Ebola and COVID-19 [15]. Addressing these environmental factors is essential for promoting a One Health approach that recognizes the interconnectedness of health across all species and ecosystems.

The socioeconomic impacts of climate change further complicate the One Health landscape. Vulnerable populations often bear the brunt of climate-related health challenges, facing food insecurity, water scarcity, and increased exposure to diseases. These stressors can lead to a decline in public health infrastructure and resources, making it more difficult to respond effectively to health crises [51]. Promoting resilience and adaptability in communities through education and collaborative initiatives is vital for mitigating these impacts and fostering a sustainable future that aligns with One Health principles.

### Impact of One Health Education on Children’s Climate Change Advocacy (2010–2023)

One Health education has significantly enhanced children’s advocacy of climate change from 2010 to 2023 by fostering a holistic understanding of the interconnections between human, animal, and environmental health. Programs that integrate One Health principles into curricula to empowered children to recognize the effects of climate change on biodiversity, health, and ecosystems. Through experiential learning activities, such as community projects and interactive lessons, children have developed critical thinking skills and a sense of agency, enabling them to engage in discussions about sustainability and advocate for environmentally friendly practices. This educational approach has not only increased knowledge but also inspired proactive attitudes towards addressing climate challenges, ultimately shaping informed future citizens committed to environmental stewardship.

### Educational Initiatives Focused on Climate Change and Its Health Implications

Educational initiatives focused on climate change and its health implications aim to raise awareness and equip individuals with the knowledge needed to understand the complex relationships between climate change, environmental health, and public health outcomes. These programs often include workshops, online courses, and interactive community events that highlight how climate change affects air quality, water resources, food security, and the spread of infectious diseases. Integrating science, policy, and health education, these initiatives encourage critical thinking and empower participants to advocate for sustainable practices and climate-resilient strategies [5, 8]. Through hands-on activities and real-world case studies, learners are motivated to take action and make informed decisions that promote both personal and community health in the context of a changing climate. Some of the educational initiatives focused on climate change and its health implications have been summarized in Table 4.

**TABLE 4:** Educational initiatives focused on climate change and its health implications (Worldwide, 2023–2025).

Initiative	Description	References
Curriculum Integration	Integrating climate change topics into health and science curricula to explore the interconnectedness of environmental changes and public health outcomes. Lessons can address air pollution, heat waves, and vector-borne diseases	[14, 22]
Community-Based Programs	Initiatives such as workshops, seminars, and public health campaigns that educate community members about climate change and its health risks. Collaborations with local health departments and NGOs can promote sustainable practices and reduce carbon footprints	[17]
Technology and Digital Platforms	Utilizing online courses, webinars, and interactive simulations to enhance outreach. Virtual reality experiences can illustrate the impacts of climate change on health, fostering deeper understanding and encouraging participation in climate action	[15]

### Community Engagement and Policy Support

Community engagement is a vital component in addressing public health issues and implementing effective One Health strategies. By actively involving community members in health initiatives, policymakers can ensure that the interventions are culturally relevant and tailored to the specific needs of the population [52]. Engagement fosters trust and collaboration, allowing for the exchange of knowledge and resources between local communities and health professionals. Furthermore, community engagement can enhance awareness and education about health risks, leading to more informed decision-making and community-led initiatives that promote healthier behaviors and practices [53].

Policy support is equally critical in facilitating the success of community-driven health initiatives. Effective policies that prioritize One Health principles can create an enabling environment for communities to thrive [54]. Policymakers must consider the input of community members when developing policies to ensure they address local realities and challenges. Incorporating community perspectives, policies can be more responsive and adaptive, ultimately leading to improved health outcomes [55]. Additionally, policies that promote interdisciplinary collaboration among sectors, such as health, agriculture, and environmental management, can strengthen the overall impact of One Health initiatives within communities.

Moreover, successful community engagement and supportive policy frameworks are essential for sustainable health interventions. When communities are empowered to participate in decision-making processes, they are more likely to take ownership of health initiatives, leading to greater sustainability and long-term success [56]. Policies that allocate resources for community capacity building, education, and training can further enhance community resilience, enabling them to respond effectively to health challenges [19]. By fostering strong partnerships between communities and policymakers, One Health initiatives can be more effectively implemented, ensuring that both local and global health priorities are met.

### Policy Recommendations for Supporting One Health Education Initiatives

Integrating One Health into national education curricula is essential for preparing future professionals to address the interconnectedness of human, animal, and environmental health. By embedding One Health principles into existing educational frameworks, students can develop a comprehensive understanding of health issues and their broader implications. Enhancing funding for One Health programs is equally important, as it enables the development of innovative research, training initiatives, and community outreach efforts that can effectively address pressing health challenges [19].

By systematically assessing the impact of One Health education and programs, stakeholders can identify best practices and areas for improvement [22]. This evidence-based approach allows for the continuous refinement of strategies, ensuring that they remain responsive to emerging health threats and changing community needs. Together, these policies create a robust framework for advancing One Health education and practice, ultimately with the aim to improved health outcomes for people, animals, and the environment. Considering the need for one health education today, some policies to this effect have been summarized on Table 5.

**TABLE 5:** Policy recommendations for supporting One Health education initiatives (Worldwide, 2022–2025).

Policy recommendation	Description	Citations
Integrate One Health into National Education Curricula	Governments should incorporate One Health principles into national education curricula across various levels, from primary to tertiary education. This integration can be achieved by developing interdisciplinary modules that combine human health, veterinary science, and environmental studies. Formalizing One Health education ensures that students gain a holistic understanding of health issues and the interconnectedness of ecosystems	[57, 58]
Enhance Funding for One Health Programs	Increased funding is essential to support the development and implementation of One Health education initiatives. Policymakers should allocate resources to educational institutions, NGOs, and community organizations that promote One Health Education programs. This funding can facilitate teacher training, the development of educational materials, and the organization of community outreach programs	[54]
Encourage Interdisciplinary Collaboration	Policies should promote collaboration among various sectors, including health, agriculture, environmental management, and education. Establishing partnerships between educational institutions and local health departments, veterinary schools, and environmental organizations can create a comprehensive approach to One Health education. Collaborative projects can help students understand real-world applications	[59] https://www.napractice.org
Support Community Engagement Initiatives	Policymakers should encourage and fund community engagement initiatives that focus on One Health. These initiatives can empower local communities to participate in health decision-making processes and promote awareness of the interconnectedness of human, animal, and environmental health. Workshops and public forums can help communities share their perspectives and foster ownership	[53]
Evaluate and Adapt Policies Based on Evidence	Continuous assessment and evaluation of One Health education programs are crucial for ensuring their effectiveness and relevance. Policymakers should establish mechanisms for monitoring and evaluating the impact of One Health initiatives on student learning and community health outcomes. Policies should be adapted based on findings to meet the evolving needs of communities	[22, 60]

### Conclusion

Successful implementation of One Health education initiatives hinges on a multifaceted approach that integrates policy recommendations and funding across various sectors. By incorporating One Health principles into national curricula, enhancing funding for educational programs, encouraging interdisciplinary collaboration, supporting community engagement, and ensuring continuous evaluation of policies, governments can foster a holistic understanding of the interconnectedness of human, animal, and environmental health. This comprehensive strategy not only equips future professionals with the necessary skills to tackle complex health challenges but also empowers communities to actively participate in health decision-making processes. Ultimately, these efforts will contribute to improved health outcomes and greater resilience in addressing global health issues.

## Footnotes

1
https://wadem.org/about/association-overview/

2
https://www.seaohun.org

3
https://isohaonehealth.wordpress.com

4
https://ohlaic.org/es/

5
http://www.getafrica.org/projects/ongoing-projects/GET-One-Health-School-Project

6
https://www.onehealthcommission.org/en/news/global_one_health_happenings_news_notes/

7
https://www.onehealthcommission.org/en/resources__services/whos_who_in_one_health/

8
https://www.uaf.edu/onehealth/education/edx.php

9
https://onehealthlessons.org/tickborne_lessons
